# Thermodynamic cycles in Josephson junctions

**DOI:** 10.1038/s41598-019-40202-8

**Published:** 2019-03-01

**Authors:** Francesco Vischi, Matteo Carrega, Pauli Virtanen, Elia Strambini, Alessandro Braggio, Francesco Giazotto

**Affiliations:** 1 NEST, Istituto Nanoscienze-CNR and Scuola Normale Superiore, I-56127 Pisa, Italy; 2 Dipartimento di Fisica, Università di Pisa, I-56127 Pisa, Italy

## Abstract

A superconductor/normal metal/superconductor Josephson junction is a coherent electron system where the thermodynamic entropy depends on temperature and difference of phase across the weak-link. Here, exploiting the phase-temperature thermodynamic diagram of a thermally isolated system, we argue that a cooling effect can be achieved when the phase drop across the junction is brought from 0 to *π* in a *iso*-*entropic* process. We show that *iso*-*entropic* cooling can be enhanced with proper choice of geometrical and electrical parameters of the junction, i.e. by increasing the ratio between supercurrent and total junction volume. We present extensive numerical calculations using quasi-classical Green function methods for a short junction and we compare them with analytical results. Interestingly, we demonstrate that phase-coherent thermodynamic cycles can be implemented by combining *iso*-*entropic* and *iso*-*phasic* processes acting on the weak-link, thereby engineering the coherent version of thermal machines such as engines and cooling systems. We therefore evaluate their performances and the minimum temperature achievable in a cooling cycle.

## Introduction

Recently, a great interest focussed on the study of mesoscopic hybrid devices with superconducting elements, both from a fundamental and technological point of view. Important applications of these systems can be found in the development of new single photon detectors^1–3^, fast and precise thermometers^4–7^, sensitive magnetometers^8–11^, low-temperature phase-coherent caloritronics^12–17^ and cryogenic machines^18–24^. To this end a deep understanding of thermodynamic aspects of mesoscopic hybrid structures is timely and of upmost importance.

Moreover, low-temperature applications, micro-refrigerators operating at sub-Kelvin range are keypoint both for quantum technology and for astrophysics purposes^25^. While it is rather easy to cool down a system until few Kelvin, investigation of system at lower temperature is not a trivial task. Today the most common approach to achieve temperature of the order of tens of mK uses adiabatic demagnetization or dilution fridges, which however have several limiting factors such as their bulky nature, their multi-stage operation and their very high-cost. Alternative cooling schemes are thus required to bypass these bottlenecks. Several systems at the microscale have been proposed to implement thermal machines, i.e. by using quantum dots^26^, single ions^27^, microelectromechanical systems^28^, piezoelectric elements^29^, or by exploiting adiabatic magnetization of superconducting structures^30,31^.

State-of-the-art cooling microsystems have been realized in hybrid structures, using a normal metal/insulator/superconductor (NIS) junction^20,32^. Once voltage biased, they can be used as cooler or heater, resulting in substantial electronic cooling power if operated in the optimal regime with a voltage very close to the superconducting energy gap. The operating principle of these systems relies on the presence of the superconducting gap in order to remove the most energetic quasiparticles from the system^20,33–35^. The performance of these systems have been further improved by connecting two junctions in series to realize a SINIS structure^36–38^.

In this work, we investigate the possibility to achieve cooling with a SNS Josephson weak-link. Differently from SINIS structures, here the mechanism relies on proximity effect^39–41^, which takes place when a normal metal is in good electric contact with a superconductor. This allows to develop a phase-tunable minigap in the quasi-particle Density of States (DoS) of the normal region^42–45^, when the weak-link is shorter or comparable to the superconducting coherence length *ξ*. This results in a non-trivial phase dependence of thermodynamic quantities, such as the entropy or the specific heat of the SNS junction. Here we exploit this phase dependence in an isolated system to envision a quantum iso-entropic process. Within this process the electronic temperature in the junction will be driven by the phase difference in order to conserve the entropy. In particular, we will show that it is possible to realize a cooling process based on this principle, similar to the adiabatic magnetic cooling^30,31^.

The thermal isolation necessary for the iso-entropic process requires low operating temperatures of our cooler in order to decouple the electronic system from the phonon bath^19,20,46^, as already demonstrated in many advanced nanotechnologies^37,47,48^.

Our phase-tunable iso-entropic process defines a new quantum thermodynamic building-block that can implement novel thermodynamic cycles. As an example we will combine two iso-entropic with two iso-phasic processes to realise a *thermodynamic Josephson cycle*. We therefore investigate in details the performances of realistic thermal machines based on this quantum coherent cycle both as engine and cooler.

The paper is organised as follows. In Section 2 we introduce the model, the basic definitions of thermodynamic quantities, the numerical and analytical approach to the proximization. In Section 3 we show the possibility of performing an iso-entropic thermodynamic process in a SNS Josephson junction, investigating its performances. Iso-entropic and iso-phasic processes are combined to form a thermodynamic Josephson cycle, as we will discuss in Section 4. Finally, Section 5 contains some comments on possible experimental realizations, while Section 6 summarizes our main findings.

## Model and thermodynamic quantities

**Figure 1:**
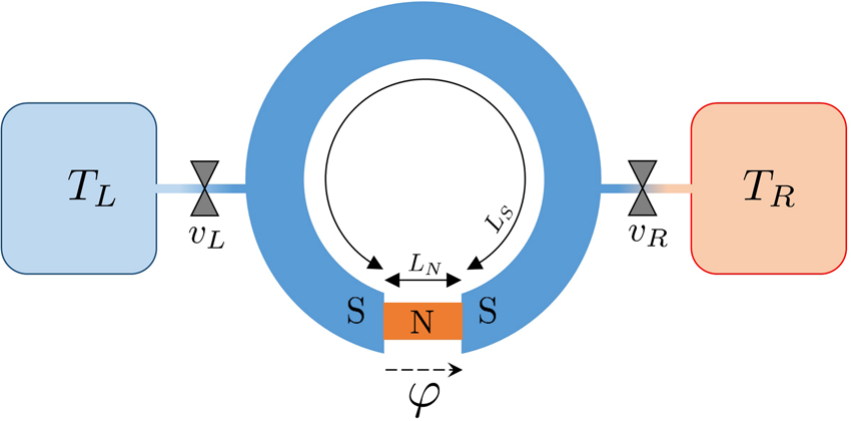
Thermodynamic SNS system. A superconductor/normal metal/superconductor Josephson weak-link is phase-biased at phase $\phi$ by a superconducting ring pierced by a magnetic flux. The electronic system can be connected via two thermal valves *v*_*L*_ and *v*_*R*_ to two external reservoirs residing at temperature *T*_*L*_ and *T*_*R*_, respectively. *L*_*N*_ and *L*_*S*_ are respectively the junction and ring lengths.

We consider a superconductor/normal metal/superconductor (SNS) Josephson weak-link phase-biased by a superconducting ring pierced by an external magnetic flux, as schematically depicted in Fig. 1. The volume of the system is $V={A}_{N}{L}_{N}+{A}_{S}{L}_{S}$ (where *A*_*S*/*N*_ and *L*_*S*/*N*_ represent the cross sectional area and the length of the superconducting/normal metal regions, respectively). In the following, we also denote with *σ*_*S*/*N*_ the associated conductivities and with Δ_0_ the superconducting energy gap at $T=0$.

We assume that this hybrid system is thermally isolated. Its electronic degrees of freedom can be connected to two reservoirs residing at temperature *T*_*L*_, *T*_*R*_ via two ideal thermal valves *v*_*j*_ with $j=L,R$, see Fig. 1. Ideal thermal valve means that they are assumed to be instantaneous, non dissipative, without thermal losses and with negligible thermal resistance in conductive state. We notice that a great effort in the research in the field of mesoscopic caloritronics^12,49–53^ is currently devoted to reach these conditions, developing novel schemes for improved thermal isolation. In the following, we assume the valves closed (no heat exchange), except for Section 4 where the valves are exploited to realise thermodynamic cycles.

### Quasi-classical theory

In general, an hybrid system consisting of a superconductor and a normal metal in electric contact shows a different behaviour of both the thermodynamic and transport properties with respect to their bulk nature in the disconnected case. This modification has been dubbed in literature under the generic name of proximity effect and is due to the propagation of the electronic correlations from the superconductor to the normal metal.

The proximity effect for dirty metals can be described within the quasi-classical theory of superconductivity^44,45^. In this framework, transport and statistical properties in thermodynamic equilibrium can be obtained by the momentum-averaged retarded Green function ${\hat{g}}^{R}(x,\varepsilon )$, a matrix defined in the electron-hole (Nambu) space, dependent on position *x* and energy *ε*^45^. The Green function ${\hat{g}}^{R}(x,\varepsilon )$ can be determined by solving the so-called Usadel equations.

We treat the SNS junction within the quasi 1-D approximation^45,54^, neglecting the edge-effects at the SN interfaces. This approximation is valid when ${A}_{S}\ll {\xi }^{2}$ or as long as the junction resistance is concentrated in the normal region^55^. In this approximation, the Usadel equations read^44,56^

1 $${\xi }^{2}{{\rm{\partial }}}_{x}({\hat{g}}^{R}(x,\varepsilon ){{\rm{\partial }}}_{x}{\hat{g}}^{R}(x,\varepsilon ))-\frac{1}{{{\rm{\Delta }}}_{0}}[-i\varepsilon {\hat{\tau }}_{3}+\hat{{\rm{\Delta }}}(x),{\hat{g}}^{R}(x,\varepsilon )]=0$$

2 $${\rm{\Delta }}(x)=\frac{\lambda }{4i}\,{\int }_{-{E}_{c}}^{{E}_{c}}\,\tanh \,(\frac{\varepsilon }{2T})\,{[{\hat{g}}^{R}(x,\varepsilon )-{\hat{g}}^{A}(x,\varepsilon )]}_{12}{\rm{d}}\varepsilon $$

where *ξ* is the superconducting coherence length^45^, *τ*_*j*_ is the j-th Pauli matrix, $\hat{{\rm{\Delta }}}(x)={\rm{\Delta }}(x){\tau }_{+}+{{\rm{\Delta }}}^{\ast }(x){\tau }_{-}$ and ${\tau }_{\pm }=({\tau }_{1}\pm i{\tau }_{2})/2$ and Δ(*x*) is the complex order parameter calculated^44^ self-consistently from ${\hat{g}}^{R}$, ${[{\hat{g}}^{R}-{\hat{g}}^{A}]}_{12}$ is the anomalous component of the Green function^44^ and ${\hat{g}}^{A}$ is the advanced Green function that can be obtained from ${\hat{g}}^{R}$ at thermal equilibrium^45^. *λ* and *E*_*c*_ define respectively the coupling constant and the energy-band of the electron-electron interaction. In practice, *λ* and *E*_*c*_ are eliminated by using standard prescription for the cut-off regularization of BCS theory^57^.

The Eq. (1) are complemented by the following boundary conditions. One of the which represents the pseudo-normalisation ${({\hat{g}}^{R})}^{2}={\mathbb{I}}$. Moreover, matching conditions^45,58^ hold at the S/N interfaces. At the position *x*_*SN*_ of the left interface (and in analogue way for the right interface), we impose the continuity

3 $${{\hat{g}}^{R}(x,\varepsilon )|}_{x\to {x}_{SN}^{-}}={{\hat{g}}^{R}(x,\varepsilon )|}_{x\to {x}_{SN}^{+}}$$

and conservation of the matrix current^58^

4 $$\frac{{\sigma }_{S}{A}_{S}}{{\sigma }_{N}{A}_{N}}{[{\hat{g}}^{R}(x,\varepsilon ){\partial }_{x}{\hat{g}}^{R}(x,\varepsilon )]}_{x\to {x}_{SN}^{-}}={[{\hat{g}}^{R}(x,\varepsilon ){\partial }_{x}{\hat{g}}^{R}(x,\varepsilon )]}_{x\to {x}_{SN}^{+}}.$$

For convenience, we rename the prefactor as a dimensionless parameter $a\equiv ({A}_{S}{\sigma }_{S})/({A}_{N}{\sigma }_{N})$ which takes into account geometric and electrical characteristic contributions of the S and N regions on proximity effect. In the following, simulations are carried with different values of *a*.

At a distance *L*_*S*_/2 from the interfaces, we set the BCS bulk boundary conditions

5 $${\hat{g}}^{R}(x={x}_{SN}-{L}_{S}/2,\varepsilon )={\hat{g}}_{{\rm{BCS}}}^{R}(\varepsilon )$$

where ${\hat{g}}_{{\rm{BCS}}}^{R}(\varepsilon )$ is given by the homogeneous case of Eq. (1). This boundary conditions has physical validity when ${L}_{S}/2\gg \xi$, since the superconductor is assumed to be long enough that the inhomogeneity effects near the junction are negligible, recovering a standard bulk form. In the following we fix ${L}_{S}=10\,\xi$.

Moreover, we focus on a SNS junction in short regime, i.e. ${L}_{N}\ll \xi$. In this regime, proximity effects are enhanced^59^ and the numerical result can be compared with analytical ones from the literature. We fix therefore ${L}_{N}=0.1\xi$ in the following.

In order to calculate the entropy of the system, we extract from the Green function the quasi-particle normalised local DoS^44,54^ by using

6 $$N(x,\varepsilon ,T,\phi )=\frac{1}{2}{\rm{Re}}\,[{\rm{tr}}\,[{\tau }_{3}{\hat{g}}^{R}(\varepsilon ,x,\phi )]]$$

The proximity effect^41,44^ alters the DoS of both the weak-link and the superconductor. Qualitatively, in the normal metal appears an induced minigap $\mathop{{\rm{\Delta }}}\limits^{ \mbox{-} }(\phi )$ whose energy width can be tuned^42–45^ by the phase difference $\phi$. In a SNS junction, the minigap decreases monotonically by increasing the phase difference from $\phi =0$ up to $\phi =\pi$, where it completely closes $\mathop{{\rm{\Delta }}}\limits^{ \mbox{-} }(\phi =\pi )=0$. Notice that $\mathop{{\rm{\Delta }}}\limits^{ \mbox{-} }(\phi )$ is symmetric for $\phi \to -\,\phi$ and 2*π*-periodic, as usually happens for time-reversal symmetric proximized Josephson systems.

Since the DoS is phase-dependent, the quasiparticle entropy $S(T,\phi )$ of the junction acquires both a temperature *T* and phase drop $\phi$ dependence. The total entropy can be expressed as $S(T,\phi )={\sum }_{j=S,N}\,{A}_{j}\,\int \,d{x}_{j}{\mathscr{S}}({x}_{j},T,\phi )$, where *x*_*j*_ denotes the curvilinear coordinate along the superconducting ring (*x*_*S*_) and normal region (*x*_*N*_). ${\mathscr{S}}$ is the quasiparticle entropy density given by

7 $${\mathscr{S}}(x,T,\phi )=-\,4{{\mathscr{N}}}_{0}\,{\int }_{-\infty }^{+\infty }\,N(x,\varepsilon ,T,\phi )f(\varepsilon ,T)\,\mathrm{ln}\,f(\varepsilon ,T){\rm{d}}\varepsilon ,$$

where $f(\varepsilon ,T)=1/({e}^{\varepsilon /T}+1)$ is the Fermi distribution function (we set $\hslash ={k}_{{\rm{B}}}=1$), $N(x,\varepsilon ,T,\phi )$ is the normalised local DoS that quantifies local variations due to the proximity effect, *N*_0_ is the normal DoS density at Fermi level.

**Figure 2:**
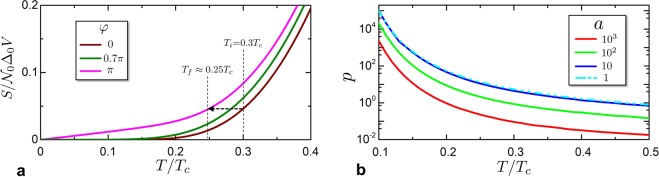
Temperature dependence of the entropy of the SNS system. (**a**) Entropy $S(T,\phi )$ as a function of temperature *T* (in units of the critical temperature *T*_*c*_) for three representative values of phase. The arrow shows an iso-entropic process from $\phi =0$ to $\phi =\pi$ and the corresponding electronic temperature variation from *T*_*i*_ to *T*_*f*_. In this plot the proximity parameter $a=({\sigma }_{S}{A}_{s})/({\sigma }_{N}{A}_{N})=100$. (**b**) Relative entropy variation *p* as a function of *T*/*T*_*c*_ for different values of *a*.

Since the entropy is given by the quasi-particle occupation of the available states, it increases from $\phi =0$ (gapped state) to $\phi =\pi$ (gapless state), as depicted in Fig. 2(a). An analysis of the phase-modulation of the entropy for proximized SNS Josephson junctions can be found in refs^59–61^, where the relations between supercurrent, entropy and inverse proximity effect are taken into account.

### Analytical Kulik-Omel’yanchuk limit

The entropy variation on phase and the supercurrent properties of the SNS junction are linked by a Maxwell thermodynamic relation^59^. Since both the entropy $S(T,\phi )$ and the supercurrent $I(T,\phi )$ can be obtained as two different derivatives of the free energy of the system^59,62^, the following identity holds:

8 $$\frac{\partial S(T,\phi )}{\partial \phi }=-\,\frac{1}{2e}\frac{\partial I(T,\phi )}{\partial T},$$

where *e* denotes the electron charge. This identity is directly due to the equilibrium nature of the Josephson current, and it establishes an exact thermodynamic relation between the excited states (quasi-particles), responsible for the entropy, and the ground state properties characterized by the Cooper pairs, responsible for the supercurrent flow.

Equation (8) implies that the entropy can be written as

9 $$S(T,\phi )={S}_{0}(T)+\delta S(T,\phi ),$$

being *S*_0_(*T*) the entropy at $\phi =0$ and

10 $$\delta S(T,\phi )=-\,\frac{1}{2e}\,{\int }_{0}^{\phi }\,\frac{\partial I(T,\phi ^{\prime} )}{\partial T}{\rm{d}}\phi ^{\prime} .$$

We consider now a particular analytical limit, given by the Kulik-Omel’yanchuk (KO) theory^55,63^. Assuming a diffusive SNS junction with short weak link (${L}_{N}/\xi \ll 1$) and resistance concentrated in the weak link ($a\to \infty$), the quasi 1-D Usadel equations can be solved analytically. From the obtained Green function it is possible to extract the current-phase relation ${I}_{{\rm{KO}}}(T,\phi )$^55,63,64^ with critical supercurrent at zero temperature given by

11 $${I}_{c}=1.32\frac{\pi {{\rm{\Delta }}}_{0}}{2e{R}_{N}}=1.32\pi \frac{{\xi }^{2}{A}_{N}}{{L}_{N}}e{{\mathscr{N}}}_{0}{{\rm{\Delta }}}_{0}^{2}.$$

The second equality is obtained by considering that ${R}_{N}={L}_{N}/({\sigma }_{N}{A}_{N})$ and that in diffusive transport it holds^45,54^ ${\sigma }_{N}=2{e}^{2}{{\mathscr{N}}}_{0}{{\rm{\Delta }}}_{0}{\xi }^{2}$.

In KO limit, the entropy at $\phi =0$ is ${S}_{0}(T)=V{{\mathscr{S}}}_{{\rm{BCS}}}(T)$ where ${{\mathscr{S}}}_{{\rm{BCS}}}$ is the homogeneous BCS entropy density, obtained by substituting in Eq. (7) the normalised BCS DoS:

12 $$N(T,\varepsilon )=|{\rm{Re}}\frac{\varepsilon }{\sqrt{{\varepsilon }^{2}-{{\rm{\Delta }}}^{2}(T)}}|.$$

For $T\lesssim 0.1\,{T}_{c}$, the entropy *S*_0_ has the asymptotic form^65,66^

13 $${S}_{0}(T\to 0)\simeq \sqrt{2\pi }{(\frac{{{\rm{\Delta }}}_{0}}{T})}^{1/2}{e}^{-{{\rm{\Delta }}}_{0}/T}V\,{{\mathscr{N}}}_{0}{{\rm{\Delta }}}_{0}.$$

The entropy variation in the KO theory is obtained^59^ by using Eq. (10) with the CPR expression obtained in KO theory^64^

14 $$\delta S(T,\phi )=-\,\tfrac{2{{\mathscr{N}}}_{0}{{\rm{\Delta }}}_{0}V\alpha }{\pi }\tfrac{\partial }{\partial T}\,{\int }_{{\rm{\Delta }}(T)\cos (\frac{\phi }{2})}^{{\rm{\Delta }}(T)}\,\tanh (\tfrac{\varepsilon }{2T})\,\mathrm{log}[\tfrac{{\rm{\Delta }}(T)|\sin (\tfrac{\phi }{2})|+\sqrt{{\varepsilon }^{2}-{\rm{\Delta }}{(T)}^{2}\,{\cos }^{2}(\tfrac{\phi }{2})}}{\sqrt{{\rm{\Delta }}{(T)}^{2}-{\varepsilon }^{2}}}]\,{\rm{d}}\varepsilon ,$$

where in the prefactor we have introduced the dimensionless parameter

15 $$\alpha \equiv \frac{e{R}_{0}{I}_{c}}{1.32{{\mathscr{N}}}_{0}{{\rm{\Delta }}}_{0}^{2}V},$$

with $${R}_{0}=\pi /{e}^{2}\approx 12.9$$ kΩ.

A linear-in-temperature behaviour can be found for $\delta S(T\to 0,\phi =\pi )$ of Eq. (14). At $\phi =\pi$ we have

16 $$\delta S(T,\phi =\pi )={{\mathscr{N}}}_{0}{{\rm{\Delta }}}_{0}V\frac{\alpha }{2\pi }{\int }_{0}^{{{\rm{\Delta }}}_{0}}\frac{\varepsilon }{{T}^{2}}{\sec }^{2}(\frac{\varepsilon }{2T})\mathrm{ln}(\frac{{{\rm{\Delta }}}_{0}+\varepsilon }{{{\rm{\Delta }}}_{0}-\varepsilon }){\rm{d}}\varepsilon ,$$

and expanding the logarithm, we obtain

17 $$\delta S(T\to \mathrm{0,}\pi )\simeq {{\mathscr{N}}}_{0}TV\frac{\alpha }{\pi }\,{\int }_{0}^{{{\rm{\Delta }}}_{0}/T}{z}^{2}\,{\sec }^{2}(z/\mathrm{2)}{\rm{d}}z,$$

with $z=\varepsilon /T$. The low temperature limit ${{\rm{\Delta }}}_{0}/T\to \infty$ gives

18 $$\delta S(T\to 0,\phi =\pi )\simeq \frac{2\pi }{3}\frac{e{R}_{0}{I}_{c}}{1.32{{\rm{\Delta }}}_{0}}\frac{T}{{{\rm{\Delta }}}_{0}},$$

where one can see a linear-in-temperature behaviour reminescent of the normal metal nature at $\phi =\pi$. At low temperature the entropy variation *δS* is proportional to the critical supercurrent *I*_*c*_ of the junction.

The numerical approach (from Eqs (1) to (7)) allow to calculate the entropy variation in the general case, for every value of *a*, *L*_*N*_. The analytic limit of the KO theory (from Eqs (8) to (15)) can be reproduced by numeric calculation if $a\to \infty$ and ${L}_{N}\ll \xi$. In order to compare the numerical with the analytical KO results, it needs to express the parameter *α* in terms of the parameters *L*_*N*_, *L*_*S*_, *α* of the Usadel equations. Considering the second equality in Eq. (11), we can write *α* as

19 $$\alpha ={\pi }^{2}\frac{\xi }{{L}_{N}}{(a\frac{{L}_{S}}{\xi }+\frac{{L}_{N}}{\xi })}^{-1}\approx \frac{{\pi }^{2}}{a}\frac{{\xi }^{2}}{{L}_{N}{L}_{S}}.$$

We remark that this equality holds only in the KO theory, i.e. assuming ${L}_{N}\ll \xi$ and $a\to \infty$. These justify the approximation to the right hand side. If the KO assumptions do not hold, Eq. (14) is not valid and the parameter *α* cannot be used.

### Entropy and heat capacity variation

To better appreciate the role of the *a* parameter it is convenient to introduce a quantity *p* that estimates the relative variation of the entropy induced by the phase in comparison to the phase independent part, defined as

20 $$p(T)=\frac{S(T,\phi =\pi )-S(T,\phi =0)}{S(T,\phi =0)}=\frac{\delta S(T,\phi =\pi )}{{S}_{0}(T)}.$$

The quantity *p*(*T*) is reported in Fig. 2(b). The various curves correspond to different values of $a=({\sigma }_{S}{A}_{S})/({\sigma }_{N}{A}_{N})$, representing different geometrical and material configurations.

From Eqs (13) and (18) it is straightforward to verify that the relative entropy variation $p(T)=\delta S(T,\pi )/{S}_{0}(T)$ scales like *α*. Therefore, to increase the relative entropy variation, and thus making larger the effect due to iso-entropic process, one should increase $\alpha \propto {I}_{C}/V$, by increasing the value of the critical current or by lowering the volume of the system *V*.

The adiabatic effects that we are going to study in the next sections depend on the entropy relative variation *p* and hence on the parameter *a* (and finally on *α*). From Eq. (15), obtained within KO theory, we could predict the expected scaling behaviour. In particular, the magnitude of the entropy variation scales like 1/*L*_*N*_, since the critical supercurrent scales like 1/*R*_*N*_ for short junctions. Instead, by increasing *L*_*S*_ the total volume of the device increases without substantially affecting supercurrent, with the consequence that the relative entropy variation behaves like 1/*L*_*S*_. We note that this argument does not hold for ${L}_{S}\lesssim \xi$, where BCS rigid boundary conditions are not anymore valid and hence the supercurrent magnitude can depend on *L*_*S*_. As already stated, here we neglect this situation by considering ${L}_{S}=10\,\xi$. It is important to notice, however, that the critical current-volume ratio cannot be increased at will: they are not independent, since for a small volume local self-consistent reduction^67,68^ of the pair potential Δ_0_(*x*) will appear, decreasing the supercurrent of the weak-link.

It is important to stress that Eqs (9) and (10), and their link with the current-phase-relationship, are general and rely on basic thermodynamic consistency relation, i.e. Maxwell relation. Hence, they hold true independently of the nature of the weak-link (insulating barrier, metallic weak-link, ferromagnet layer, etc.).

For a SIS junction, where the current-phase-relationship is given by the Ambegaokar-Baratoff formula^55,57^, the entropy variation at low temperature is

21 $$\delta {S}_{{\rm{AB}}}(T\to 0,\phi =\pi )=\frac{e{R}_{0}{I}_{c}}{2\pi }\frac{{{\rm{\Delta }}}_{0}}{{\cosh }^{2}(\frac{{{\rm{\Delta }}}_{0}}{2T}){T}^{2}}.$$

Comparing this result with Eq. (18), we notice that the entropy variation in a SIS is exponentially suppressed with respect to that of a SNS junction, with a completely different temperature dependence. In order to enhance the effects of an iso-entropic transformation, therefore, it is convenient to consider a SNS junction instead of a SIS one. This is due to the particular feature of the SNS junction that, thanks to the DoS of the N region, allows the phase-modulation of the correlations and transport properties over a volume of magnitude *ξ*^3^, differently to a SIS junction that concerns a zero-length insulator layer. This is the reason why in this paper we mainly concentrate on proximized SNS systems. The above discussion shows the complete generality of the presented mechanism, which can be realized with different kind of junctions or with different external thermodynamic variables opening the road to further developments in the thermodynamic characterisation of novel hybrid systems.

A comment on the heat capacity *C* of the system is now in order. This is a measurable quantity, encoding the temperature variation of the system after a given heat pulse, produced for example by Joule heating. Here, we can expect that the heat capacity is phase-dependent, being

22 $$C(T,\phi )=T\frac{\partial S(T,\phi )}{\partial T}.$$

Hence, the two different temperature behaviours of entropy at $\phi =0$ and $\phi =\pi$ are reflected in the different behaviours of the heat capacity. Notice that here we consider the heat capacity at constant phase; this quantity can be different from the heat capacity at constant flowing supercurrent, in analogy with the different heat capacity of an ideal gas at constant volume or constant pressure. From Eq. (13) we obtain that the heat capacity at $\phi =0$ is

23 $$C(T,\phi =0)=\sqrt{2\pi }{(\frac{{{\rm{\Delta }}}_{0}}{T})}^{3/2}{e}^{-{{\rm{\Delta }}}_{0}/T}V\,{{\mathscr{N}}}_{0}{{\rm{\Delta }}}_{0},$$

while the heat capacity at $\phi =\pi$ is $C(T,\phi =\pi )=C(T,\phi =0)+\delta C(T,\phi =\pi )$, where (see Eq. (18))

24 $$\delta C(T,\phi =\pi )=T\frac{\partial \delta S(T,\phi )}{\partial T}=\frac{2\pi }{3}\frac{e{R}_{0}{I}_{c}}{1.32{{\rm{\Delta }}}_{0}}\frac{T}{{{\rm{\Delta }}}_{0}}.$$

Similarly to the entropy, $$C(T,\phi =\pi )\approx \delta C(T,\phi =\pi )$$ at low temperature. Then, the heat capacity has a strongly different behaviour on *T*/Δ_0_ whether the phase is $$\phi =0$$ or $$\phi =\pi $$, respectively an exponential decrease or a linear behaviour with decreasing of temperature. Note that in the latter case one has the same linear-in-temperature behaviour expected for a normal metal, i.e. $$C=2{\pi }^{2}{{\mathscr{N}}}_{0}VT/3$$. Notice that relative heat capacity variation $$\delta C(T,\phi =\pi )/C$$$$(T,\phi =0)$$ scales with *α*. Hence, the value of *α* can be estimated by a heat capacity measurement at $$\phi =0$$ and $$\phi =\pi $$.

It is important to note that at low temperature the gapped nature of the superconducting leads exponentially suppress their heat capacity (see Eq. (23)) producing a limited contribution with respect to the proximized region, even though they have larger in volume. This is one of the reasons behind the effectivity of the proposed iso-entropic transformation in affecting the electron temperature of the total system.

## Iso-entropic process

In the previous section we have discussed the phase and temperature dependence of the thermodynamic entropy of a SNS junction. Exploiting these features, we now study the properties of an iso-entropic process in which the entropy remains constant, while externally varying the phase $\phi$ of the weak-link. In order to retain the entropy constant, this process will result in a temperature variation, in particular in a electronic temperature decrease of the junction as we will demonstrate below. To implement such a process we assume that the system is thermally isolated, and does not exchange heat with the environment and phonons (see also Section 5 for a detailed discussion of this issue in realistic experimental conditions). For this reason, in a single iso-entropic process the two thermal valves *v*_*L*_ and *v*_*R*_ sketched in Fig. 1 are closed. Exploiting a physical analogy with classical thermodynamics, this iso-entropic process is similar to an adiabatic expansion/compression of an ideal gas. In both situations there is no heat exchange with an external reservoir and the number of available states is modified by the variation of a thermodynamical variable, typically an external parameter. In the former case tuning the phase $\phi$ one can modify the value of the minigap (and consequently the DoS), while in the latter case varying the volume the available states will change.

**Figure 3:**
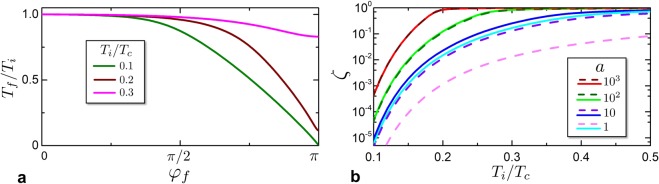
Temperature decrease in a iso-entropic process. (**a**) The relative temperature decrease *T*_*f*_ /*T*_*i*_ as a function of phase ${\phi }_{f}$ of the junction, where *T*_*i*_ is the initial temperature at ${\phi }_{i}=0$. Different curves refer to different initial temperature *T*_*i*_/*T*_*c*_ at a fixed $a=100$. (**b**) shows the ratio $\zeta ({T}_{i})={T}_{f}/{T}_{i}$ for an iso-entropic process $({T}_{i},{\phi }_{i}=0)\to ({T}_{f},{\phi }_{f}=\pi )$, as a function of initial temperature *T*_*i*_. Different curves refer to different values of *a*. Solid lines correspond to numerical solutions of Usadel equations; dashed lines correspond to analytic results obtained within the KO theory. The KO theory and the full numeric solution are in good agreement at $a={10}^{2},{10}^{3}$; deviations appear for lower values of *a*.

Let us consider the system to be in a initial thermodynamic state $({T}_{i},{\phi }_{i}=0)$ with a entropy ${S}_{i}=S({T}_{i},{\phi }_{i}=0,)$. In an iso-entropic process, the entropy remains constant, thus, in a quasi-static process that brings the phase from ${\phi }_{i}=0$ to ${\phi }_{f}$, the final temperature is determined by the entropy equation $S({T}_{f},{\phi }_{f})={S}_{i}$. Here, a relation ${T}_{f}({\phi }_{f},{S}_{i})$ between the temperature and the phase in the final state is implicitly established. In particular, since the entropy increases from ${\phi }_{i}=0$ to $0 < {\phi }_{f}\le \pi$, the isolated system will decrease its temperature from its initial value *T*_*i*_. This is shown in Fig. 3(a), where we plot the relative temperature decrease ${T}_{f}({\phi }_{f},{T}_{i})/{T}_{i}$ as a function of ${\phi }_{f}$. Notice that greater temperature decrease can be achieved for lower initial temperature *T*_*i*_ (see e.g. the curve corresponding to ${T}_{i}=0.1{T}_{c}$ in Fig. 3(a)). Recalling the symmetry properties of the supercurrent^39^ and Eq. (8), it is possible to argue that ${T}_{f}({\phi }_{f},{S}_{i})$ is 2*π*-periodic even function in ${\phi }_{f}$.

From now on we concentrate on a process that brings the junction from ${\phi }_{i}=0$ to ${\phi }_{f}=\pi$, as sketched with the black arrow in Fig. 2(a), that maximize the temperature decrease. For these two values the supercurrent flowing through the junction is zero, allowing also to neglect the contribution to the system energy of the ring inductance (this point is further clarified in Section 4). We define $\zeta (T)\equiv {T}_{f}/T$ for an iso-entropic process from $({\phi }_{i}=0,T)$ to $({\phi }_{f}=\pi ,{T}_{f})$. At low temperature (${T}_{i}\lesssim 0.2\,{T}_{c}$), *ζ*(*T*_*i*_) is characterised by an exponential decrease as a consequence of the energy mismatch between the distribution $f(\varepsilon )\,\mathrm{log}\,f(\varepsilon )$ and the proximized DoS with induced minigap $\overline{{\rm{\Delta }}}$. When $\phi$ approaches *π*, the induced minigap $\overline{{\rm{\Delta }}}$ closes and the energy window associated to $f(\varepsilon )\,\mathrm{log}\,\,f(\varepsilon )$ becomes greater than the minigap. As a consequence, the phase-dependence of the entropy integral in Eq. (7) is stronger at low temperatures. This property is reflected in the different behaviour of the entropy at $\phi =0$ and $\phi =\pi$ in Fig. 2(a).

In Fig. 3(b) we report *ζ*(*T*_*i*_) as a function of *T*_*i*_. Various curves refer to different geometries for different values of the dimensionless proximity parameter *a*. In this figure and the followings, the solid curves represent the numerical solution obtained by solving the Usadel equations^10,59^ within the specified geometry (see Section 2). The dashed lines are instead obtained within the KO theory, calculating of $S(T,\phi )$ by mean of Eqs (7), (12) and (14). In all cases, there is good agreement between the full numeric results and the KO theory for *a* = 10^2^, 10^3^, while deviations appear at lower values of *a* where KO theory overestimates the temperature decrease. This show that numerical solution of the proximized system is necessary when *a* become smaller as would be desirable in order to decrease the final temperature.

**Figure 4:**
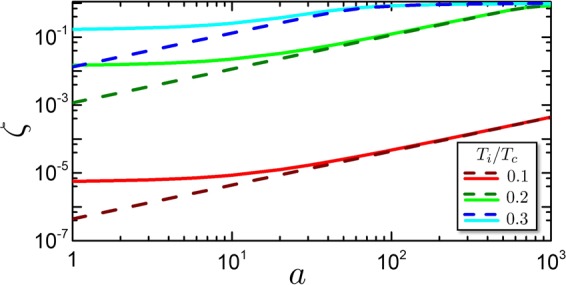
Ratio $\zeta ={T}_{f}/{T}_{i}$ as a function of *a*. Different curves refer to different initial temperature *T*_*i*_/*T*_*c*_. Solid lines are calculated by numerically solving the Usadel equations. Dashed curves are analytical solutions within the KO theory, valid in the limit $a\gg 1$.

From Fig. 3(b) one can argue that *ζ*(*T*_*i*_) grows with *a*. This is shown in detail in Fig. 4, where *ζ*(*T*_*i*_) is plotted as a function of *a* for three different values of initial temperature *T*_*i*_. When $a\gg 1$, the current-phase-relationship of the junction tend to the KO asymptotic limit ($a\to \infty$) where the supercurrent magnitude is determined only by the junction geometry. In this limit, the entropy variation has the form of Eq. (14) with a scale determined by the critical supercurrent magnitude *I*_*c*_. Furthermore increasing the volume *V* of the system, *a* increases and consequently the phase-independent contribution *S*_0_(*T*) of entropy increases. Then, the relative entropy variation *p* decreases scaling with *α*, spoiling the iso-entropic effects. In other words, the contribution *S*_0_ that increases with *V* acts as a heat-capacity that mitigate the iso-entropic temperature decrease. On the contrary, when $a\to 1$, the KO theory does not hold anymore. In particular, the current-phase-relationship is no more determined by the weak link characteristics only, but depends also on the geometrical parameters of the superconductor. This is due to the fact that the normal metal decreases the correlations in the superconductor banks, with the final result that a superconductor region nearby the SN interface behaves like a normal metal. As a consequence, the SNS junction behaves like a weak link with an effective length that is longer than the geometrical length^39,67,69^ and with a reduced supercurrent magnitude that decreases with *a*. This effect contributes to decrease the iso-entropic effects, as shown by the fact that the numerical calculation (solid lines) in Figs 3 and 4 return a worse iso-entropic temperature decrease.

The temperature decrease *ζ*(*T*_*i*_) can be obtained by solving numerically the transcendental equation ${S}_{0}({T}_{i})={S}_{0}({T}_{f})+\delta S({T}_{f},\phi =\pi )$. However for $T\to 0$ the phase-independent contribution in *S*_0_(*T*_*f*_) (with exponential behaviour in *T*_*f*_, see Eq. (13)) can be neglected respect to the linear term in Eq. (18). In this case, $S({T}_{f},\phi =\pi )\approx \delta S({T}_{f},\phi =\pi )$ and we obtain

25 $${\zeta }_{{\rm{KO}}}({T}_{i})\sim \frac{3}{\alpha \sqrt{2\pi }}{(\frac{{{\rm{\Delta }}}_{0}}{{T}_{i}})}^{3/2}{e}^{-{{\rm{\Delta }}}_{0}/{T}_{i}},$$

where the subscript KO means that the equation is obtained within the KO theory. Writing *α* explicitly, it turns out that ${\zeta }_{{\rm{KO}}}({T}_{i})\propto V/{I}_{c}$, confirming that the iso-entropic temperature decrease is enhanced by increasing the supercurrent and decreasing the volume. This is an important quantity because *ζ*(*T*_*i*_)*T*_*i*_ represents the minimum achievable temperature in this iso-entropic transformation.

## Thermodynamic cycles

In this section we exploit the above results to implement thermodynamic cycles with a phase-biased Josephson weak-link. For sake of simplicity, here we limit the discussion to a particular cycle, although different thermodynamic cycles can be implemented. We thus combine two iso-entropic with two iso-phasic processes, in which $\phi$ is kept constant. In the following, we restrict to iso-phasic curves at $\phi =0$ and $\phi =\pi$.

We investigate the Josephson Cycle properties and performances as an engine and a cooling system. These two different configurations depend on the orientation in which the processes are performed and which temperatures of the cycle are fixed by the reservoirs *T*_*L*_ and *T*_*R*_ as depicted in Fig. 1.

It is important to understand the form of the work and the heat associated to the weak-link during a certain process. We therefore fix the following sign convention: (I) the heat *Q* absorbed by the system from the environment is positive, (II) the work *W* released from the system to the environment is positive, (III) the sign of the supercurrent is positive when flowing in the direction of the phase gradient.

The work is done by an external magnetic field that induces a dissipationless current *I* on the system. This work increases the energy of the system through two components. One is due to the reversible energy stored in the inductance that we can neglect since we treat the two states at *I* = 0. The second component represents the Josephson energy stored in the junction^57^. The work done on a junction from $\phi =0$ to $\phi =\pi$ at constant temperature *T* (i.e. in a iso-thermal process) is given by $\frac{1}{2e}\,{\int }_{0}^{\pi }\,I(\phi ,T){\rm{d}}\phi$, where $I(\phi ,T)$ is the iso-thermal current-phase-relationship. In the case of an iso-entropic process, we must consider that the temperature is not constant. Considering the phase-dependence of the temperature ${T}_{f}(\phi ,{T}_{i})$ (see Fig. 3(a)), the work done by the system to the environment in an iso-entropic process $\phi =0\to \phi =\pi$ reads

26 $${W}_{i\to f}=-\,\frac{1}{2e}\,{\int }_{0}^{\pi }\,{I}_{S}(\phi ,{T}_{i}){\rm{d}}\phi ,$$

where *I*_*S*_ can be defined as

27 $${I}_{S}({T}_{i},\phi )=I(\phi ,{T}_{f}(\phi ,{T}_{i})).$$

**Figure 5:**
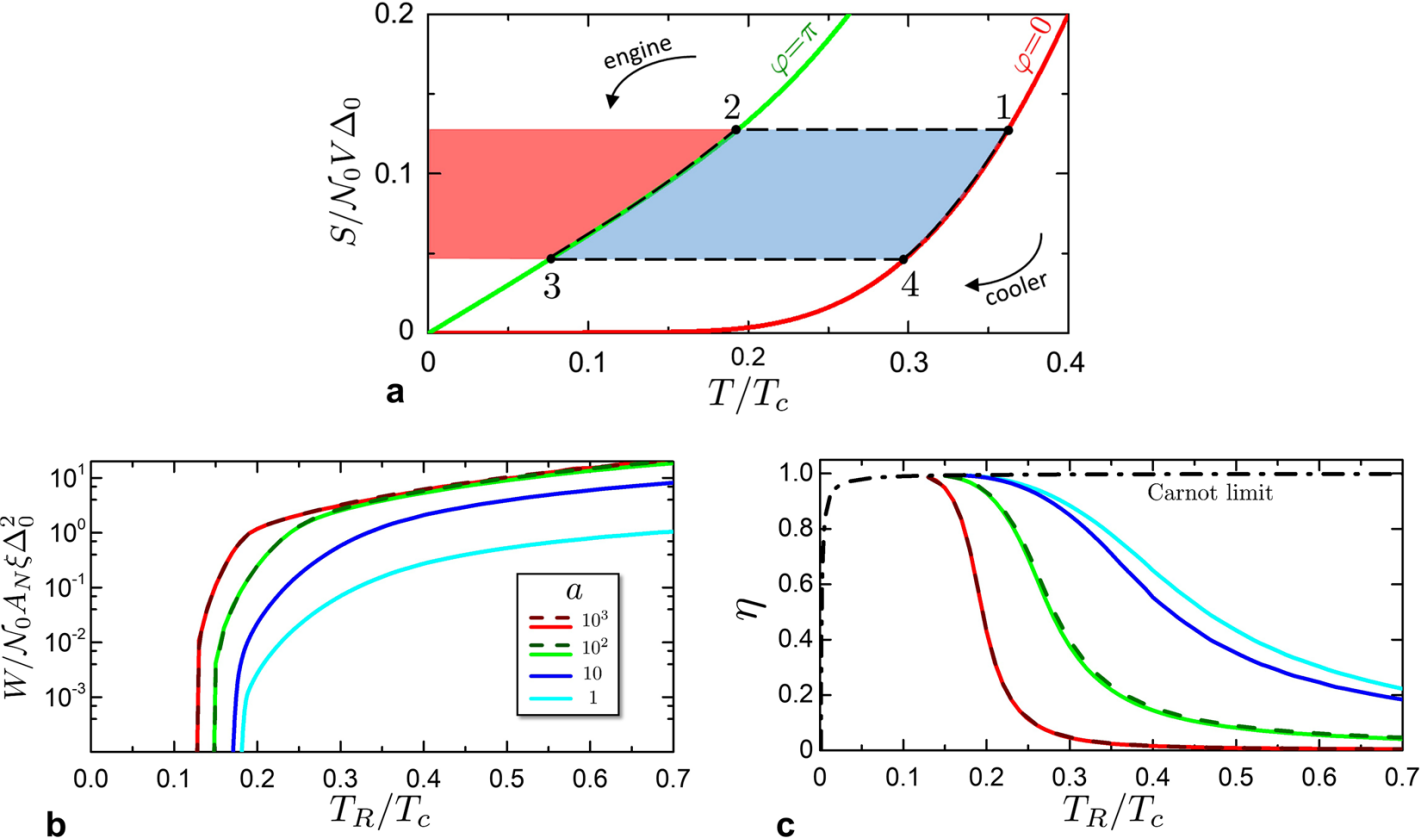
SNS system as a thermodynamic machine. (**a**) Scheme of a cycle in the entropy/temperature plane, consisting in the two iso-entropic processes 1–2 and 3–4 and the two iso-phasic processes 2–3 and 4–1. The red area represents the heat (released or absorbed) in the process 2–3. The light blue area is the work *W* (done or received) in a complete cycle. The cycle can be used as engine or cooler machine if performed respectively counter-clockwise or clockwise. (**b**) Work *W* of the engine as a function of the hot temperature *T*_*R*_ at fixed ${T}_{L}={10}^{-3}{T}_{c}$. Different curves refer to some values of *a*. Dashed curves are analytical solutions obtained within the KO theory. The curves are defined when ${T}_{R}\ge {T}_{{\rm{act}}}$. (**c**) Thermodynamic efficiency $\eta$ of the Josephson engine, for different *a*, as a function of *T*_*R*_/*T*_*c*_, with ${T}_{L}={10}^{-3}{T}_{c}$. The black dot-dashed curve represents the Carnot limit 1 − *T*_*L*_/*T*_*R*_. The curves are defined for ${T}_{R}\ge {T}_{{\rm{act}}}$.

In the following, we use the notation *W*_*jl*_ to indicate the work done by the junction in a process from the thermodynamic state *j* to the state *l*, where *j*, *l* represent two states in Fig. 5(a).

In the process $1\to 2$ the work done by the system is negative, the environment must spend an amount of energy to charge the Josephson inductance of the junction, increasing the free energy of the latter^57,70,71^. On the contrary, the system would release work when discharged from $\phi =\pi$ to $\phi =0$ (process $3\to 4$). The work in a iso-phasic process is zero, being zero the phase variation, ${W}_{23}={W}_{41}=0$.

At the same time, the heat absorbed by the junction in a process from the state *j* to the state *l* is

28 $${Q}_{jl}={\int }_{j}^{l}\,T{\rm{d}}S,$$

where $${Q}_{jl}=-\,{Q}_{lj}$$.

In the entropy/temperature plane of Fig. 5(a), the absolute value of heat *Q*_23_ is represented by the red area. The absolute value heat *Q*_41_, instead is the sum of the red and blue area. In order to calculate the net work $W={W}_{12}+{W}_{34}$ done in one cycle, we consider the conservation of energy which states that within a cycle *W* is equal to the net heat absorbed by the system $Q={Q}_{23}+{Q}_{41}$, i.e. $W=Q$. Considering that *Q*_23_ and *Q*_41_ have opposite sign, the blue area in Fig. 5(a) represents the net work *W* per cycle.

### Josephson engine

An engine is a thermodynamic machine which can convert the temperature gradient between a hot and cold reservoirs into useful work. Referring to Fig. 1, we set the cold and hot reservoirs to be at temperature *T*_*L*_ and *T*_*R*_, respectively. The cold reservoir can be though as an environment (e.g. large electric pad well-thermalised with the substrate at the base temperature of a cryostat) and the hot reservoir as a Joule heated pad with a continuous supplier of power. The Josephson engine consists of the following cycle, as sketched in Fig. 5(a):

- **Iso**-**entropic 1** → **2**. The junction is initially at temperature of the hot reservoir ${T}_{1}={T}_{R}$ and the valves *v*_*L*_, *v*_*R*_ are closed (thermally isolated junction). Then, an iso-entropic transformation brings the system from $({T}_{1}={T}_{R},{\phi }_{1}=0)$ to $({T}_{2},{\phi }_{2}=\pi )$. The junction absorb a certain amount of work |*W*_12_| with no heat exchange.
- **Iso**-**phasic 2** → **3**. The junction is put in contact with the cold reservoir by opening valve *v*_*L*_ and keeping the phase difference $\phi =\pi$. The iso-phasic transformation brings the system from $({T}_{2},{\phi }_{2}=\pi )$ to $({T}_{3}={T}_{L},{\phi }_{3}=\pi )$. The junction releases heat |*Q*_23_| to the cold reservoir, without doing any work.
- **Iso**-**entropic 3** → **4**. The valve *v*_*L*_ is closed and the junction is again thermally isolated. An iso-entropic transformation brings the system from $({T}_{3}={T}_{L},{\phi }_{3}=\pi )$ to $({T}_{4},{\phi }_{4}=0)$. The junction returns the work *W*_34_ and no heat is exchanged.
- **Iso**-**phasic 4** → **1**. The junction is put in contact with the hot reservoir by opening valve *v*_*R*_ and keeping the phase $\phi =0$. The iso-phasic transformation brings the system from $({T}_{4},{\phi }_{4}=0)$ to $({T}_{1}={T}_{R},{\phi }_{1}=0)$. The junction absorbs heat *Q*_41_ from the hot reservoir, without any work.

We fix the cold temperature to be ${T}_{L}={10}^{-3}{T}_{c}$ (magnitude of), and we study the engine performance as a function of *T*_*R*_. Once fixed the cold reservoir at *T*_*L*_, it exists a minimum temperature threshold for the hot reservoir, below which the engine does not work. This threshold coincides with *T*_4_, thus defined as activation temperature ${T}_{{\rm{act}}}={T}_{4}$. This is implicitly defined by the equation $\zeta ({T}_{{\rm{act}}}){T}_{{\rm{act}}}={T}_{L}$. When *T*_*R*_ approaches *T*_act_, ${T}_{2}\to {T}_{L}$ and ${T}_{R}\to {T}_{4}$, with the consequence that the energy stored in the junction in the process $1\to 2$ tends to the energy returned in the process $3\to 4$, and $W\to 0$. The activation temperature can be understood as follows: starting from the cycle in Fig. 5(a), by decreasing *T*_*R*_ till *T*_act_ the blue area collapses to a line, indicating that the work goes to zero.

Figure 5(b) shows the net work *W* released by the junction as a function of *T*_*R*_ for a fixed ${T}_{L}={10}^{-3}{T}_{c}$. The curves are defined for ${T}_{R}\ge {T}_{{\rm{act}}}$. When ${T}_{R}\to {T}_{{\rm{act}}}$ the *W* curves go to zero. As one would expect, at fixed *a*, the net work is an increasing function of *T*_*R*_, see that the blue area in Fig. 5(a) will increase if *T*_1_ increases. A similar argument applies for the *a* dependence of the net work, which tends to saturate for large values of *a*, while approaching the KO limit. On the other side the work decreases with decreasing *a*. The reason is that for small *a*, the effectivity of the iso-entropic transformation to change the device temperature is progressively affected as a consequence of the reduction of the relative entropy variation *p* (see discussion in Section 2). The blue area of Fig. 5 is reduced because transformation $2\to 3$ and $4\to 1$ become arbitrarily close, i.e. the neat work reduces.

Figure 5(c) reports the efficiency of the Josephson engine $\eta =W/{Q}_{41}=1-|{Q}_{23}|/{Q}_{41}$. Also here, the various curves are defined for ${T}_{R}\ge {T}_{{\rm{act}}}$. The dash-dotted black curve is the Carnot efficiency limit given by ${\eta }_{C}=1-{T}_{L}/{T}_{R}$. For ${T}_{R}\to {T}_{{\rm{act}}}$ the efficiency tends to the Carnot limit. Considering Fig. 5(a), the efficiency can be visualized as the ratio between the blue area and the total (red + blue) area. When the areas collapse to a line, the efficiency tends to $\eta =({T}_{4}-{T}_{3})/{T}_{4}=1-{T}_{3}/{T}_{4}$ that equals ${\eta }_{C}=1-{T}_{L}/{T}_{R}$. This shows, for a Josephson engine, a common property shared with other engines that when the thermodynamical efficiency is maximal then the work produced tends to zero.

Analytical results can be obtained within the KO theory in the limit where temperatures are much smaller than Δ_0_. The value of *T*_act_ can be found by solving

29 $${T}_{L}={\zeta }_{{\rm{KO}}}({T}_{{\rm{act}}}){T}_{{\rm{act}}}=\frac{3{{\rm{\Delta }}}_{0}}{\alpha \sqrt{2\pi }}{(\frac{{{\rm{\Delta }}}_{0}}{{T}_{{\rm{act}}}})}^{1/2}{e}^{-{{\rm{\Delta }}}_{0}/{T}_{{\rm{act}}}}.$$

By using Eq. (28), the heat released to the cold reservoir can be evaluated as

30 $${Q}_{23}={\int }_{{S}_{2}}^{{S}_{3}}\,T\,dS=\frac{\pi }{3}\frac{e{R}_{0}{I}_{c}}{1.32}[{(\frac{{T}_{3}}{{{\rm{\Delta }}}_{0}})}^{2}-{(\frac{{T}_{2}}{{{\rm{\Delta }}}_{0}})}^{2}],$$

where we have used Eq. (18) which governs entropy at $\phi =\pi$ for small *T*. Substituting ${T}_{2}=\zeta ({T}_{R}){T}_{R}$ and ${T}_{3}={T}_{L}$,

31 $${Q}_{23}=\frac{\pi }{3}\frac{e{R}_{0}{I}_{c}}{1.32}[{(\frac{{T}_{L}}{{{\rm{\Delta }}}_{0}})}^{2}-{(\frac{\zeta ({T}_{R}){T}_{R}}{{{\rm{\Delta }}}_{0}})}^{2}].$$

For ${T}_{L}\to 0$ using Eq. (25), we obtain that $|{Q}_{23}|\propto {T}_{R}^{2}{V}^{2}/{I}_{c}$. As expected, the released heat increases with the temperature of the hot reservoir. The quadratic behaviour in *V* is due to the fact that both the heat capacity and the temperature difference ${T}_{2}-{T}_{L}\approx \zeta ({T}_{R}){T}_{R}$ increase with volume *V*. The behaviour as 1/*I*_*c*_ is determined by the product of the heat capacity prefactor at ($\phi =\pi$), which grows with *I*_*c*_ (see Eq. 24), with the temperature squared ${T}_{2}^{2}={(\zeta ({T}_{R}){T}_{R})}^{2}\propto 1/{I}_{c}^{2}$.

The heat ${Q}_{41}={\int }_{4}^{1}\,TdS$ absorbed by the system can be easily calculated using the Laisant theorem, which shows that ${\int }_{4}^{1}\,T\,dS+{\int }_{4}^{1}\,S\,dT={S}_{1}({T}_{1}){T}_{1}-S({T}_{4}){T}_{4}$.

Finally one finds ${Q}_{41}=S({T}_{R}){T}_{R}-S({T}_{4}){T}_{4}-{\int }_{4}^{1}\,SdT$ where the last integral can be easily evaluated from the BCS free energy expression^65,66^. Anyway for ${T}_{R}\ll {{\rm{\Delta }}}_{0}$ that integral may be safely neglected due to the exponential suppression of BCS entropy. Finally one finds

32 $${Q}_{41}=\sqrt{2\pi }[{(\frac{{T}_{R}}{{{\rm{\Delta }}}_{0}})}^{1/2}{e}^{-{{\rm{\Delta }}}_{0}/{T}_{R}}-{(\frac{{T}_{4}}{{{\rm{\Delta }}}_{0}})}^{1/2}{e}^{-{{\rm{\Delta }}}_{0}/{T}_{4}}]\,V\,{{\mathscr{N}}}_{0}{{\rm{\Delta }}}_{0}^{2}.$$

The work is given by $W={Q}_{23}+{Q}_{41}$ and for ${T}_{L}\to 0$ we found

33 $$W=\sqrt{2\pi }{(\frac{{T}_{R}}{{{\rm{\Delta }}}_{0}})}^{1/2}{e}^{-{{\rm{\Delta }}}_{0}/{T}_{R}}[1-\frac{3}{2\alpha \sqrt{2\pi }}{(\frac{{{\rm{\Delta }}}_{0}}{{T}_{R}})}^{3/2}{e}^{-{{\rm{\Delta }}}_{0}/{T}_{R}}]\,V\,{{\mathscr{N}}}_{0}{{\rm{\Delta }}}_{0}^{2}.$$

This expression is valid because $S(T,\phi =\pi )\approx \delta S(T,\phi =\pi )$ in the low temperature limit. Since the second term in the square brackets is proportional to 1/*α*, the work slightly increases with supercurrent as ~$1-\kappa V/{I}_{c}$ where *κ* is a factor exponentially suppressed at low temperatures. The work follows roughly the temperature scaling ${T}_{R}^{1/2}{e}^{-{{\rm{\Delta }}}_{0}/{T}_{R}}$.

Finally, the efficiency is given by

34 $$\eta =1-\frac{3}{2\alpha \sqrt{2\pi }}{(\frac{{{\rm{\Delta }}}_{0}}{{T}_{R}})}^{3/2}{e}^{-{{\rm{\Delta }}}_{0}/{T}_{R}},$$

and it decreases with temperature and ${\alpha }^{-1}\propto V/{I}_{c}$. From this rough estimation we expect that indeed it increases while lowering *a* for a fixed *T*_*R*_, and this is indeed found in the full numerical solution plotted with solid lines in Fig. 5(c).

### Josephson cooler

We now discuss the thermodynamic cycle in the opposite configuration, i.e. acting as a cooler. Indeed, cooling can be obtained by reversing the cycle described in the previous section and by considering that the role of the reservoir is different in this situation.

We consider the junction to be connected to a reservoir at temperature *T*_*R*_ via a thermal valve *v*_*R*_, and to an external system –to be cooled– initially at temperature *T*_*L*_, named cooling-fin in the following. The latter system has to be thermally isolated from other spurious heat sources. This may be realised in nanoscaled suspended systems, such as membranes^48^, circuits^47^ and low dimensional electronic systems.

The Josephson cooling cycle consists in the following four processes

1. **4** → **3**. The junction is in equilibrium at environment temperature ${T}_{4}={T}_{R}$ and the thermal valves *v*_*L*_, *v*_*R*_ are closed. An iso-entropic transformation brings the system from $({T}_{4}={T}_{R},{\phi }_{4}=0)$ to $({T}_{3},{\phi }_{3}=\pi )$ doing work over the junction.
2. **3** → **2**. The thermal valve *v*_*L*_ is open, making the junction in contact with the cooling fin, reaching *T*_*L*_. In this process, the junction absorb heat *Q*_32_ from the cooling fin.
3. **2** → **1**. The thermal valve *v*_*L*_ is closed. An iso-entropic transformation brings the system from $({T}_{2}={T}_{L},{\phi }_{2}=\pi )$ to $({T}_{1},{\phi }_{1}=0)$. In this process, work is released from the junction to the external circuit and no heat is exchanged. The temperature *T*_1_ represents the higher temperature of the cycle and is the analogue of the temperature in the heat exchanger in refrigerators.
4. **1** → **4**. The thermal valve *v*_*R*_ is open, making the junction in contact with the environment. The junction temperature is lowered from *T*_1_ to *T*_*R*_, releasing heat to the environment.

The temperature *T*_3_ is again determined by the iso-entropic cooling from the temperature of the environment ${T}_{4}={T}_{R}$. The temperature *T*_3_ is the minimum possible temperature of the cooling-fin, i.e. the minimum achievable temperature ${T}_{{\rm{MAT}}}({T}_{R})$ once given *T*_*R*_ as the one of the hot reservoir. The *T*_MAT_ is given by an iso-entropic transformation starting from the state $({T}_{R},\phi =0)$ to $({T}_{{\rm{MAT}}},\phi =\pi )$. We thus recall analytic result for *T*_MAT_ that holds at ${T}_{R}\ll {{\rm{\Delta }}}_{0}$ in the KO theory:

35 $${T}_{{\rm{MAT}}}={\zeta }_{{\rm{KO}}}({T}_{R}){T}_{R}=\frac{3{{\rm{\Delta }}}_{0}}{\alpha \sqrt{2\pi }}{(\frac{{{\rm{\Delta }}}_{0}}{{T}_{R}})}^{1/2}{e}^{-{{\rm{\Delta }}}_{0}/{T}_{R}}.$$

If ${T}_{L}={T}_{{\rm{MAT}}}$, no heat can be absorbed by the junction from the cooling-fin.

**Figure 6:**
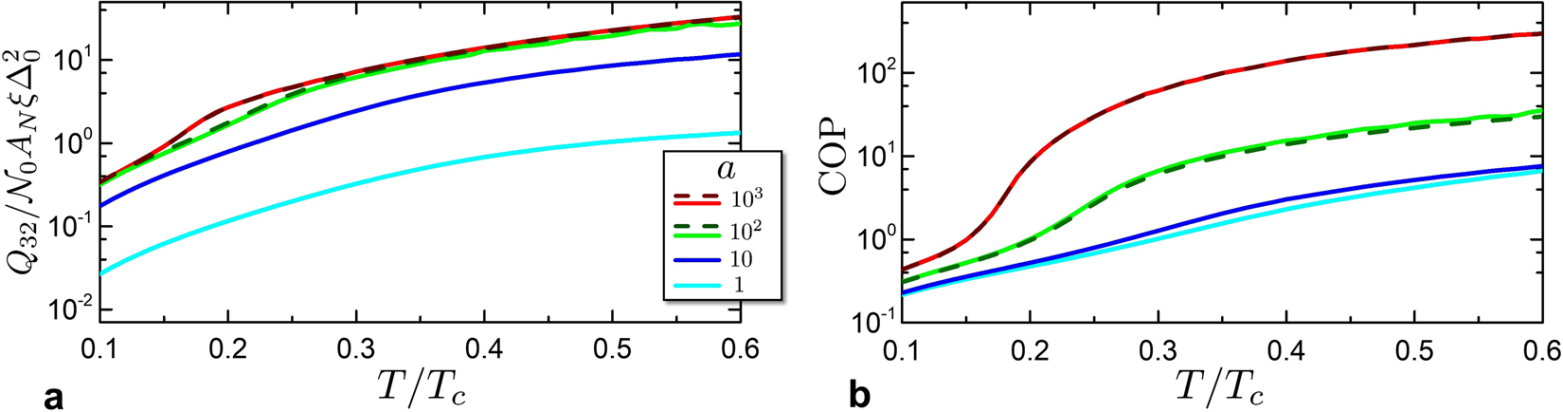
Josephson cooler characteristics. (**a**) Cooling Power per cycle *Q*_32_ of the refrigerator at cooling fin temperature equal to the environment temperature ${T}_{L}={T}_{R}$, as a function of *T*_*R*_/*T*_*c*_ and for different values of *a*. Solid curves are obtained by numerical solution while dashed curves represent analytical results obtained within the KO theory. (**b**) Coefficient of performance of the cooler at ${T}_{R}={T}_{L}$ for various *a*.

To characterize the performance of the Josephson cooler, we discuss the cooling power per cycle *Q*_32_ and the coefficient of performance COP of the SNS junction when the cooling-fin temperature equals the temperature of the environment, i.e. ${T}_{L}={T}_{R}=T$. In this state the cooling power is maximum and decreases to zero when the cooling-fin temperature approaches the *T*_MAT_. The cooling power per cycle *Q*_32_ represents the amount of heat removed from the cooling-fin and is plotted in Fig. 6(a). Indeed the iso-entropic cooling process reduces the device temperature lower than the cooling fin and by thermalization the heat is absorbed by the cooler from the cooling-fin. The cooling power then depends on the heat capacity of the system during the thermalisation process (3 → 2 in Fig. 5). When the system passes from the state 4 to the state 2 with the same temperature *T* with the phase difference tuned from 0 to *π*, a certain amount of heat must be absorbed by the system, since the heat capacity is increased by $\delta C=T{\partial }_{T}\delta S$ (see Section 3). This quantity scales with the supercurrent magnitude and for $a\to \infty$ converges to a function defined by the KO current-phase relationship. This quantity can be estimated for $T\ll {{\rm{\Delta }}}_{0}$ as

36 $${Q}_{32}=\frac{\pi }{3}\frac{e{R}_{0}{I}_{c}}{1.32}(1-{\zeta }_{{\rm{KO}}}{(T)}^{2})\frac{{T}^{2}}{{{\rm{\Delta }}}_{0}^{2}}.$$

The dependence on *a* is encoded in *ζ*_KO_(*T*). Neglecting the second term in the square brackets (since $\zeta (T)\to 0$ for $T\to 0$), the cooling power per cycle is

37 $${Q}_{32}=\frac{\pi }{3}\frac{e{R}_{0}{I}_{c}}{1.32}\frac{{T}^{2}}{{{\rm{\Delta }}}_{0}^{2}}.$$

Intriguingly this limit does not depend on *a* but only on the junction critical supercurrent. Looking at Fig. 6(a), one see from the full numerical solution (solid lines) that *Q*_32_ increases with *a* and they tend to saturate to a particular limit for large values of *a*. This trend is a consequence of the limited value of the critical current in the KO limit ($a\to \infty$). Indeed the cooling power increases with *a* due to the fact that the junction effective lengths decrease when approaching the KO limit with maximal critical current^39,67,69^.

The coefficient of performance (COP) is defined as the ratio of the pumped heat per cycle with the work spent per cycle, i.e. ${\rm{COP}}=|{Q}_{32}|/W$. We note that in analogy with the efficiency $\eta$ of an engine, also for this quantity there is a maximal limit COP_*C*_ that depend on the temperature of the two reservoirs, given by ${{\rm{COP}}}_{C}={T}_{L}/({T}_{R}-{T}_{L})$. In the specific case we are considering here with ${T}_{R}={T}_{L}$ the COP_*C*_ diverges, therefore any finite value of COP may be considered as a sort of inefficiency of the cooler with respect to an ideal one.

In Fig. 6(b) we show the COP as a function of the working temperature. One immediately see that the maximal performance of the cooler is obtained for $a\to \infty$. This corresponds to the maximal value of the absorbed work. At the same time the work absorbed per cycle decreases with $a\to \infty$. The reason is the following: increasing *a*, ${T}_{1}\to {T}_{4}$ and ${T}_{3}\to {T}_{2}$. Hence, the iso-entropic electric work −*W*_43_ done on the system tend to *W*_21_, with the consequence that the net work per cycle $W={W}_{43}+{W}_{21}$ tend to zero. For this reason, the COP increases with *a*, as reported in Fig. 6(b).

It is possible to evaluate an asymptotic expression of the COP as a function of the temperature *T*_1_ by solving the implicit equation $T={\zeta }_{{\rm{KO}}}({T}_{1}){T}_{1}$. In the following calculation, we took for *Q*_32_ the estimate of Eq. (37) and for *Q*_14_ we apply the same approach used to derive Eq. (32) but neglecting the contribution of *T* since in the limit $T\to 0$ one has $T\ll {T}_{1}$. So the heat exchange of the system with the hot reservoir is

38 $${Q}_{14}=-\,\sqrt{2\pi }{(\frac{{T}_{1}}{{{\rm{\Delta }}}_{0}})}^{1/2}{e}^{-{{\rm{\Delta }}}_{0}/{T}_{1}}V\,{{\mathscr{N}}}_{0}{{\rm{\Delta }}}_{0}^{2},$$

where the minus sign indicates that the heat is released by the system to the reservoir.

This rough estimates allows to inspect the scaling properties of the cooling cycle COP on *T*_1_ and *α*. The COP can be written as

39 $${\rm{COP}}=-\,\frac{{Q}_{32}}{{Q}_{32}+{Q}_{14}}=\frac{1}{|\frac{{Q}_{14}}{{Q}_{23}}|-1},$$

where

40 $$|\frac{{Q}_{14}}{{Q}_{23}}|=\alpha \frac{{(2\pi )}^{3/2}}{3\pi }{(\frac{{T}_{1}}{{{\rm{\Delta }}}_{0}})}^{3/2}{e}^{{{\rm{\Delta }}}_{0}/{T}_{1}}.$$

Finally we have

41 $${\rm{COP}}=\frac{1}{2\sqrt{2\pi }\alpha {({T}_{1}/{{\rm{\Delta }}}_{0})}^{3/2}{e}^{{{\rm{\Delta }}}_{0}/{T}_{1}}/3-1}.$$

At low temperatures the COP behaves as ${\alpha }^{-1}{({T}_{1}/{{\rm{\Delta }}}_{0})}^{-3/2}{e}^{-{{\rm{\Delta }}}_{0}/{T}_{1}}$ so, it decreases with temperatures decreasing. At the same time we expect to see that it grows with *a* as suggested by the dependence of *α*, i.e. Eq. (15). All these behaviours are indeed seen in Fig. 6(b) where the solid lines represent the full numerical results and the dashed lines the results obtained within KO theory. Interestingly, increasing the supercurrent the COP decreases as 1/*I*_*c*_. The reason is that, increasing the supercurrent, the work scales as a power greater than the cooling power per cycle. This is due to the enhanced adiabatic decreasing of temperature. Therefore, improving the cooling power will result in a minor coefficient of performance, a common feature shared by refrigerators.

## Possible experimental implementations

Here, we clarify which are the main physical requirements that need to be satisfied in order to realise the proposed thermal machines and we give some estimations on the expected performances.

The first important issue concerns the possibility to realise an iso-entropic transformation, i.e. to thermally isolate the electron system of the SNS junction from the thermal bath for the time necessary to perform the transformation. In these metallic system, electrons thermally relax mainly by electron-electron and electron-phonon interactions, with respectively the two characteristic time scales *τ*_*e*-*e*_ and *τ*_*e*−*ph*_. An efficient iso-entropic process should be faster than the electron-phonon relaxation time and slower than the electron-electron relaxation time, keeping the electron system in thermal quasi-equilibrium. This condition can be achieved in typical superconductors, where the two time scales are well-separated at low temperature, as demonstrated in several experiments^19,20,46,72^. Moreover, at temperatures below $T/{T}_{c}\approx 0.175$, the *τ*_*e*−*e*_ tends to saturate^73^, meanwhile *τ*_*e*−*ph*_ is expected to be exponentially suppressed^74,75^. The general reason for ${\tau }_{e-e} < {\tau }_{e-ph}$ is given by the fact that electron relaxation can mediated by many channels, among these also the phononic channel^76^.

In particular, a superconductor with a high ratio ${\tau }_{e-ph}/{\tau }_{e-e}$ is the aluminium^72^: ${\tau }_{e-e}$ ~ 1–10 ns and ${\tau }_{e-ph}$ ~ 1–10 μs at *T* ~ *T*_*c*_. Lowering the temperature, at $T\,\lessapprox \,0.1\,{T}_{c}$, the ${\tau }_{e-e}$ saturates^76^ to 10^2^–10^3^ μs. A similar phenomenon has been observed in tantalum^76^, with ${\tau }_{e-e}$ saturating to 10^1^–10^2^ μs. Experiments in titanium nitride^73^ or niobium nitride^77^ show respectively ${\tau }_{e-ph}\sim 100\,\,{\rm{ns}}$ and ${\tau }_{e-ph}\sim 200\,\,{\rm{ps}}$, suggesting that these material can be used at higher frequencies. Depending on the specific materials, iso-entropic processes are thus possible with operating frequencies that can vary from 1–10 kHz (aluminium or tantalum) to 0.01–10 GHz (NbN or TiN). We notice that equilibration times increase at low temperatures, so one needs experimentally to find a good trade-off between the validity of the iso-entropic hypothesis and equilibration times in those systems. Investigate how the maximal operating frequency is affected by material selection, specific system design, operating temperature and non-idealities of the thermal valves is beyond the target of this paper.

In view of possible experimental realizations, we can give some estimates on the expected performance of the proposed thermal machines, based on state-of-the-art materials and experimental parameters. The cooling power is given by Eq. (37), where ${R}_{0}=h/2{e}^{2}\approx 12.9$ kΩ, $e=1.602\times {10}^{-19}\,{\rm{C}}$. At $T=0.2\,{T}_{c}$ (corresponding to $kT=0.11\,{{\rm{\Delta }}}_{0}$), supercurrent ${I}_{c}=1\,{\rm{mA}}$ and operating frequency $\nu =100\,\,{\rm{MHz}}$, we obtain a cooling power $\dot{Q}={Q}_{32}\nu \sim 2\,{\rm{pW}}$.

The work can be estimated from Fig. 5b, where at $T=0.2{T}_{c}$ it is $W={A}_{N}\xi {{\mathscr{N}}}_{0}{{\rm{\Delta }}}_{0}^{2}$. To express this quantity as function of *I*_*c*_, we refer to Eqs (15) and (19), yielding ${A}_{N}\xi {{\mathscr{N}}}_{0}{{\rm{\Delta }}}_{0}^{2}=e{R}_{0}{I}_{c}/130$. At ${I}_{c}=1\,{\rm{mA}}$ and $\nu =100\,\,{\rm{MHz}}$ we obtain again $\dot{W}=W\nu \sim 2\,{\rm{pW}}$.

A possible implementation for thermal valves can be realized with quantum point contacts realized on top of two dimensional electron gas, offering the high tunability and the degree of thermal isolation required to test our predictions^78–81^. In this case good thermal contact between the two dimensional electron gas and the superconductor ring can be achieved using InAs-based quantum well and Nb or Al as superconductors^78–82^ providing very high transparencies of the interfaces. A rough estimate of the expected temperature reduction for a realistic setup where the cooling-fin is done by a two-dimensional electron gas can be done.

In III–V semiconductor crystals at sub-Kelvin temperatures the electron-phonon piezoelectric coupling is the dominant process for the heat exchange between electrons and the environment^83,84^. Then, for a realistic setup in which the cooling fin is made by a quantum well, the heat transferred by the hot phonons to the electrons can be written as ${\dot{Q}}_{e-ph}(T,{T}_{ph})={\rm{\Sigma }}{\mathscr{A}}({T}_{ph}^{5}-{T}^{5})$ with ${\mathscr{A}}$ the area of the quantum well and *T* and *T*_*ph*_ are the electron and phonon temperatures, respectively. The coupling constant^84^ has the typical values of ${\rm{\Sigma }}\approx 30$ fW μm^−2^ K^−5^. The equilibrium electronic temperature T* of the cooling-fin can be easily obtained by solving the heat balance equation ${\dot{Q}}_{e-ph}({T}^{\ast },{T}_{ph})={\dot{Q}}_{{\rm{cool}}}({T}^{\ast })$ where the cooling power of the system is ${\dot{Q}}_{{\rm{cool}}}(T)={Q}_{32}(T)\nu$ with *W*_32_ taken from equation (37) and *ν* is the operating frequency of the cooling cycle. Using the previous values and assuming for the cooling-fin the area ${\mathscr{A}}\approx 100$ μm^2^ with a phonon temperature of ${T}_{ph}\approx 100\,{\rm{mK}}$ one finds for the equilibrium electron temperature $T\approx 1\,\,{\rm{mK}}$. If $T\ll {T}_{ph}$ the solution of the balance equation may be approximated with $T\approx \sqrt{{T}_{ph}^{5}/\beta }$ with $\beta =(\pi /3)\,(e{R}_{0}{I}_{c}/1.32)\,(\nu /{{\rm{\Delta }}}_{0}^{2}{\rm{\Sigma }}{\mathscr{A}})$. This estimation would not take in account of many non-idealities such as non-ideal point contact thermal valve or limits due to intrinsic diffusive time. Those limitations need to be carefully addressed at the design stage of the proposed device and this goes beyond the scope of the present work.

This discussion shows the potential of this cooling cycle and demonstrates that the proposed thermodynamic cycles could operate efficiently in the sub-Kelvin regimes playing an important role for many different quantum technology platforms.

## Summary and Conclusions

In this work, we have considered the thermodynamic properties of a proximized SNS Josephson junction in the diffusive regime. We have shown that the phase- and temperature-dependent entropy can be exploited to achieve significant temperature decrease of the electronic degrees of freedom of the system. In particular, one can implement iso-entropic processes, by externally tuning the phase drop of the weak-link, getting temperature variations consistent with thermodynamic constraints. Elaborating on this concept, we have demonstrated the possibility to build thermodynamic cycles based on the combination of iso-entropic and iso-phasic processes. By coupling the SNS junction to two thermal baths via two thermal valves, we have shown that it is possible to engineer a Josephson engine and cooler by coherently driving the phase across the weak-link. We have studied in detail these thermal machines, investigating their performances such as the efficiency or the cooling power as a function of different geometrical and electrical parameters. Full numerical calculations have been supported by asymptotic calculations valid in the short junction regime within the KO theory, discussing several limiting behaviours. We have also proposed a possible experimental setup to implement the discussed device as powerful cooler at sub-Kelvin regimes.
